# LncRNA SNHG1 functions as a ceRNA to antagonize the effect of miR‐145a‐5p on the down‐regulation of *NUAK1* in nasopharyngeal carcinoma cell

**DOI:** 10.1111/jcmm.13497

**Published:** 2018-03-25

**Authors:** Xintang Lan, Xiuling Liu

**Affiliations:** ^1^ Department of Otolaryngology Head and Neck Surgery Weihai Municipal Hospital Weihai China

**Keywords:** nasopharyngeal carcinoma, SNHG1, miR‐145‐5p, *NUAK1*, EMT

## Abstract

How lncRNA SNHG1 influences the aggressiveness of nasopharyngeal carcinoma cells as well as the underlying mechanism was studied. The lncRNA differences were analysed by GSE12452 gene microarray. The expression of SNHG1, MiR‐145‐5p and *NUAK1* was identified by qRT‐PCR and western blot. Transfection was conducted to construct nasopharyngeal carcinoma cells with different expressions of SNHG1, miR‐145‐5p and *NUAK1*. Dual‐luciferase reporter assay was performed to explore the relationship between SNHG1, miR‐145‐5p and *NUAK1*. Wound‐healing assay and transwell invasion experiments were employed to study changes in cell migration capacity and cell invasion, respectively. Tumour xenografts were performed to observe lung metastasis of nude mice inoculated with transfected CNE cells. SNHG1 is highly expressed in nasopharyngeal carcinoma tissues and in cell lines. Down‐regulation of SNHG1 facilitated the expression of miR‐145‐5p and further suppressed the level of NAUK1 in CNE and HNE‐1 cells. Silencing of SNHG1, up‐regulation of miR‐145‐5p and inhibition of NAUK1 by relative transfection all attenuated the aggressiveness of CNE and HNE‐1 cells both *in vivo* and *in vitro*. Moreover, the impaired cell migration and invasion by SNHG1 siRNA could be rescued by cotransfection of miR‐145‐5p in CNE and HNE‐1 cells. LncRNA SNHG1 promoted the expression of *NUAK1* by down‐regulating miR‐145‐5p and thus promoted the aggressiveness of nasopharyngeal carcinoma cells through AKT signalling pathway and induced epithelial‐mesenchymal transition (EMT).

## Introduction

Nasopharyngeal carcinoma (NPC) generates in nasopharyngeal epithelium. It is one of the most popular malignant head and neck neoplasms 1. Neck lymph node metastasis and distant metastasis have frequently been seen in NPC 2, making NPC therapy challenging. Currently, radiotherapy and chemotherapy are commonly used in NPC treatment 3. A large variety of patients have shown radioresistance and chemoresistance, yet little is known about the molecular mechanism of resistance. However, to better understand the mechanism especially the drug resistance mechanism may not only help with effective treatment of NPC but also contribute to the deeper comprehension of NPC development.

Long non‐coding RNAs (lncRNAs), with more than 200 nucleotides, are transcriptional RNA molecules; lncRNAs have limited or non‐protein‐coding capacity 4. Many lncRNAs have been found to play vital roles in tumorigenesis and progression 5. In addition, previous studies have shown that the lncRNA expression patterns varied significantly between NPC tissues and normal nasopharyngeal tissues 6. LncRNA SNHG1 was frequently overexpressed in stomach cancer tissues, and its overexpression was reported to be associated with the propagation of gastric cancer cells 6. Down‐regulation of SNHG1 inhibited colorectal carcinogenesis 7. SNHG1 could also encourage cell propagation in glioma 8, prostate cancer 9 and non‐small cell lung carcinoma (NSCLC) 10, *etc*. However, studies on the functional relevance of lncRNAs in NPC are quite a few.

MicroRNAs (miRNAs) are endogenous ∼22‐nucleotide RNAs, regulating genes by pairing to the mRNAs of protein‐coding genes 11. The occurrence of many human cancers is connected with miRNAs and lncRNAs, whose abnormal expression or function disorder is found in many neoplasms 12. Some miRNAs including miR‐145‐5p play important role in the diagnosis and treatment of diverse human neoplasms 13. For example, miR‐145‐5p reduced melanoma cell propagation and aggressiveness 14. Clinically, the expression of miR‐145‐5p was closely associated with lymph node metastasis in NSCLC 9. Down‐regulation of miR‐145‐5p was also correlated with poor prognosis of gastric cancer 15.

Novel (nua) kinase family (NUAK) 1 (also known as ARK5) belongs to AMP‐activated protein kinase catalytic subunit family. The activation of *NUAK1* is directly affected by Akt, which is important in tumour malignancy 16. *NUAK1* has close relationship with cancer development and patients’ survival rate. For instance, *NUAK1* correlated with poor prognosis of ovarian cancer 17. MiR‐204 inhibited human NSCLC metastasis through inhibiting *NUAK1* 18. MiR‑96 suppressed tumour growth by targeting *NUAK1* in pancreatic cancer 19. However, few of the studies have connected *NUAK1* with miRNAs or lncRNAs in NPC.

We intended to study how SNHG1 affects NPC development. We have been suggested that SNHG1 might regulate *NUAK1* expression *via* miR‐145‐5p and thereby modulate NPC development. Our study could be a valuable reference in the development of therapeutic strategy against human NPC.

## Materials and methods

### Tissue samples and cell lines

Tissue samples were selected from participants in the NPC case‐control study conducted by Weihai Municipal Hospital. Twenty‐seven cases were histologically diagnosed as NPC tumour samples, and 9 were biopsy negative control (NC). Informed consents were collected from all participants. This study has gained the approval of the ethics boards of the Weihai Municipal Hospital. Cell lines HEK293T (the human embryonic kidney cell line), N69, CNE and HNE‐1 were purchased from BeNa Culture Collection (BNCC).

### Gene expression microarray

Differentially expressed lncRNAs were screened out from GSE12452 profile (https://www.ncbi.nlm.nih.gov/geo/). Nine normal nasopharyngeal tissues and 27 NPC tissues were included for searching the differentially expressed lncRNAs. The filtration criteria were as follows: Log_2_ (Fold Change) > 2 and *P* < 0.05.

### RT‐qPCR

TRIzol and PureLink RNA Mini Kit were purchased from Thermo Fisher Scientific (Waltham, MA, USA). They were used to isolate total RNA from cells or tissues. RNA was reverse transcribed into cDNA using TIANScript II RT Kit (Tiangen, Beijing China). RealMasterMix (SYBR Green) (Tiangen) was used for quantitative PCR assays. *GAPDH* was the internal reference. Each experiment was repeated at minimum thrice. ABI7500 quantitative PCR instrument was used for RT‐qPCR assays. The primer (Sangon Biotech, Shanghai, China) sequences used were as Table 1.

**Table 1:** Primer sequences in RT‐qPCR

cDNA	Primer sequences
GAPDH‐F	5′‐AAGGTGAAGGTCGGAGTCA‐3′
GAPDH‐R	5′‐GGAAGATGGTGATGGGATTT‐3′
SNHG1‐F	5′‐AGGCTGAAGTTACAGGTC‐3′
SNHG1‐R	5′‐TTGGCTCCCAGTGTCTTA‐3′
NUAK1‐F	5′‐TGCAGGCTGGAGATCCTACT‐3′
NUAK1‐R	5′‐TTCTAGACGGCAGGTCAGGT‐3′
miR‐145‐5p‐F	5′‐CAGTGCGTGTCGTGGAGT‐3′
miR‐145‐5p‐R	5′‐AGGTCCAGTTTTCCCAGG‐3′
U6 –F	5′‐CTTCGGCAGCACATATAC‐3′
U6 –R	5′‐GAACGCTTCACGAATTTGC‐3′

### Cell culture and transfection

NP69, CNE, HNE1 and CNE‐2Z cell lines were cultured in RPMI‐1640 media with 10% foetal bovine serum (FBS). HEK293T cells were cultured in Dulbecco's modified media (DMEM) media with 10% FBS. HONE‐1 cell line was cultured in 90% high‐glucose DMEM with 10% FBS. RPMI‐1640 and DMEM media were purchased from GIBCO BRL (Grand Island, NY, USA). All cell culture was maintained in a humidified and 5% CO_2_ sterile incubator. The si‐SNHG1 (si‐SNHG1‐1, si‐SNHG1‐2 and si‐SNHG1‐3), si‐NC, miR‐145‐5p mimics, miR‐NC mimics, miR‐145‐5p inhibitor as well as NC inhibitor were synthesized by Gene Pharma (Shanghai, China). These small molecules were transfected into cells using Lipofectamine 3000 (Invitrogen, Carlsbad, CA, USA). Transfection efficiency was measured 48 hrs later. Cells in mock group underwent no treatment.

### Western blot

Proteins were extracted from cells using RIPA buffer (Solarbio, Shanghai, China). The extracted proteins went through 12% sodium dodecyl sulphate polyacrylamide gel electrophoresis (SDS‐PAGE) and transferred to 0.22 μm PVDF blotting membranes (Millipore, Billerica, MA, USA) *via* constant voltage 80V electroporation. The membranes were blocked for 1 hr with FBS at room temperature. After that, the membranes were hatched with primary antibodies overnight at 4°C. Primary antibodies against NUAK1, Akt, p‐Akt, E‐cadherin, N‐cadherin, MT1‐MMP, MMP‐2, MMP‐9 and GAPDH were used in this experiment (primary antibodies were purchased from Cell Signaling Technology, Danvers, MA, USA). Membranes were subsequently washed with TBST (TBS, 1 ml/l Tween‐20) the following day after 30‐min. room temperature rewarming and incubated with antirabbit IgG‐horseradish peroxidase secondary antibodies for 2 hrs at a dilution of 1:1000 at room temperature and finally washed for 10 min. Immunoreactivity was visualized with an ECL Western blotting detection kit (GE Healthcare, Amersham, UK) using X ray. Densitometric analysis was performed with Image Lab software (Bio‐Rad, Hercules, CA, USA).

### MTT assay

At 48 hrs after transfection, cells were harvested and plated in 96‐well plates at a concentration of 5 × 10^4^ per well. After an additional culture for 48 hrs, a total of 20 μl 3‐(4, 5‐dimethylthiazol‐2‐yl)‐2, 5‐diphenyltetrazolium bromide (MTT) was added into each well, and cells were incubated for another 4 hrs. Thereafter, the formazan crystals were dissolved with the addition of 150 μl DMSO. Absorbance at 490 nm was detected, and relative cell viability was calculated.

### Dual‐luciferase reporter assay

The sequence of SNHG1 containing the miR‐145‐5p binding site was cloned into the psiCHECK2, therefore the constructed vector was named as SNHG1‐WT; correspondingly, the luciferase reporter SNHG1‐Mut was constructed containing the mutated sequence at miR‐145‐5p binding site. Similarly, *NUAK1* 3′ UTR cDNA containing the intact miR‐145‐5p recognition site was amplified and was subcloned into luciferase reporter psiCHECK2, finally designated as *NUAK1*‐UTR‐WT. Correspondingly, the luciferase reporter *NUAK1*‐UTR‐Mut was generated. The 100 ng WT, Mut or empty psiCHECK2 reporter plasmids were cotransfected with 50 pmol miR‐145‐5p mimics or miR‐NC mimics into HEK293T cells. After 48 hrs culture, the transfected cells were gathered. Luciferase activities in each group were measured using Dual‐Luciferase Reporter Assay kit (Promega, Shanghai, China).

### Wound‐healing assay

Cells were allowed to grow in 6‐well plates and confluence in normal complete media with 10% FBS. Wounds on monolayers of every well were made using pipettes when the cells grew to approximately 50% confluence. Then, the medium was carefully changed to fresh RPMI 1640 medium containing 10% FBS. Monolayers of each well were photographed and evaluated 12 and 24 hrs after the wound creation. Each experiment was repeated for at minimum 3 times.

### Transwell assay

Transwell assay cells were pre‐incubated, collected and resuspended in serum‐free RPMI‐1640 medium. 100 μl cell suspension (1 × 10^5^ cells/ml) was placed in the matrigel‐coated upper chamber. The wells below were filled with 0.75 ml RPMI‐1640 media with 10% FBS. Cells were allowed to invade for 36 hrs, after which the cells left on the upper side of filters were removed. The cells on the bottom side of the filters were stained with 5% crystal violet. The filters were photographed, and the number of violet cells was counted under a microscope.

### Animal experiments

Sixteen 4‐week‐old female BALB/c nude mice were purchased from Guangdong Medical Laboratory Animal Center (Guangzhou, China). The nude mice experiments were approved by the Animal Experimental Ethics Committee of Weihai Municipal Hospital. To establish a xenograft tumour model of lung colonization, 1 × 10^6^ per 200 μl of PBS of CNE cells that stably transfected with agomiR‐145‐5p, sh‐SNHG1 or sh‐NUAK1 (provided by Yunkelong, Wuhan, China) or untreated cells was injected into the tail veins of the mice (*n* = 4/group, 16 in total). After 6 weeks of growth, the mice were killed, and the lung tissues were harvested, weighed and cut into 5 mm tissue sections for histological validation.

### HE staining

Lung tissues of mice (16 in the xenograft tumour model of lung colonization) were dewaxed using xylene for 10 min. twice; the tissues were washed with ethanol for 5 min. twice, then immersed in 100%, 95%, 80%, 70% and 50% ethanol for 2 min., respectively. Next, they were washed in running water for 1 min. and distilled water for 1 min. Stained with haematoxylin for 5 min. and differentiated in 1% hydrochloric acid alcohol for 20 sec. (microscopic control), the tissues were washed in running water for 2 min. After the dehydration in 50%, 70% and 80% alcohol for 2 min., respectively, tissues were treated with 1% ammonium hydroxide, eosin for 2 min., 95%, 100%, 100% ethanol for 2 min., respectively, and xylene for 10 min. twice. Finally, tissues were sealed with neutral resin.

### Statistical analysis

All measured data were documented as average ± standard deviation. Statistics were analysed with SPSS statistics 24.0 (SPSS, Inc, Chicago, IL, USA). Student's *t*‐test and one‐way anova were adopted to analyse the normally distributed data and GraphPad Prism 6.0 (GraphPad Software, San Diego, CA, USA) was used to draw the diagrams. The differences between/among groups were considered statistically significant if *P <* 0.05.

## Results

### SNHG1 expression was high in NPC tissues and cell lines

**Figure 1:**
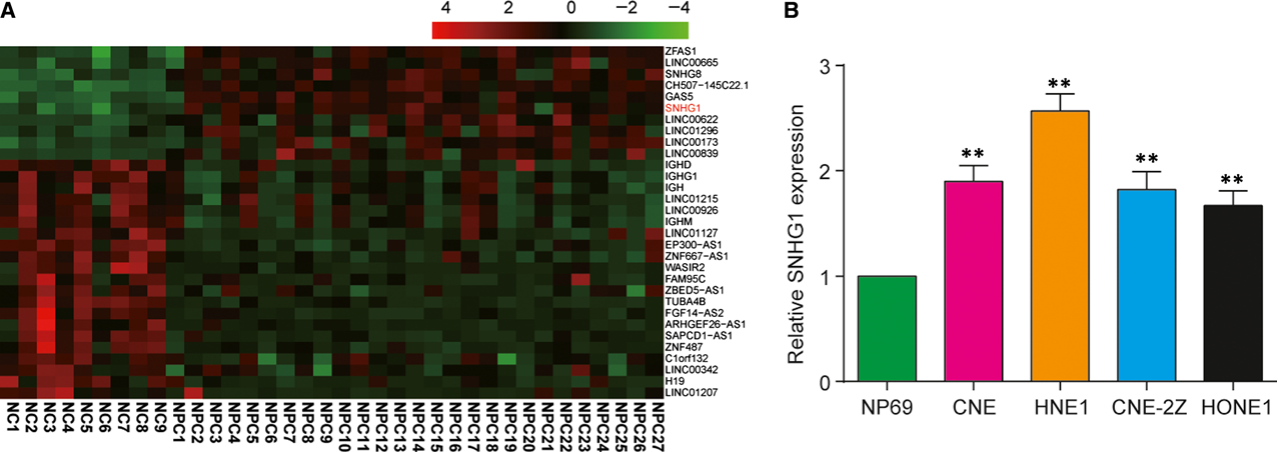
The high expression levels of SNHG1 in nasopharyngeal carcinoma. (**A**) Different expression of lncRNAs in 27 nasopharyngeal carcinoma tissues (NPC) and 9 normal nasopharyngeal tissues (NC). The gene expression profile was normalized and visualized by Genesis software. Red represents high expression, and green represents low or absent expression. (**B**) The relative expression levels of SNHG1 were higher in NPC cell lines (CNE, HNE1, CNE‐2Z and HONE1) than that in normal nasopharyngeal epithelial cell line (NP69) measured by qRT‐PCR. Number of normal tissues = 9, number of NPC tissues = 27. **P < 0.01 compared with NP69 group.

Compared with the 9 normal tissues, the expression level of SNHG1 in 27 NPC tissues increased by 1.02 times (*P <* 0.01). There were 10 up‐regulated and 21 down‐regulated lncRNAs in NPC tissues. The expression profile was shown in Figure 1A. The expression of SNHG1 in CNE, HNE1 CNE‐2Z and HONE1 cell lines was higher than that in NP69 cell line (*P <* 0.05). Among the four cancer cell lines, CNE and HNE‐1 showed the highest SNHG1 level (Fig. 1B). These findings indicated that SNHG1 demonstrated aberrantly high level in NPC cell lines.

### MiR‐145‐5p was the target of SNHG1 and was suppressed by SNHG1

**Figure 2:**
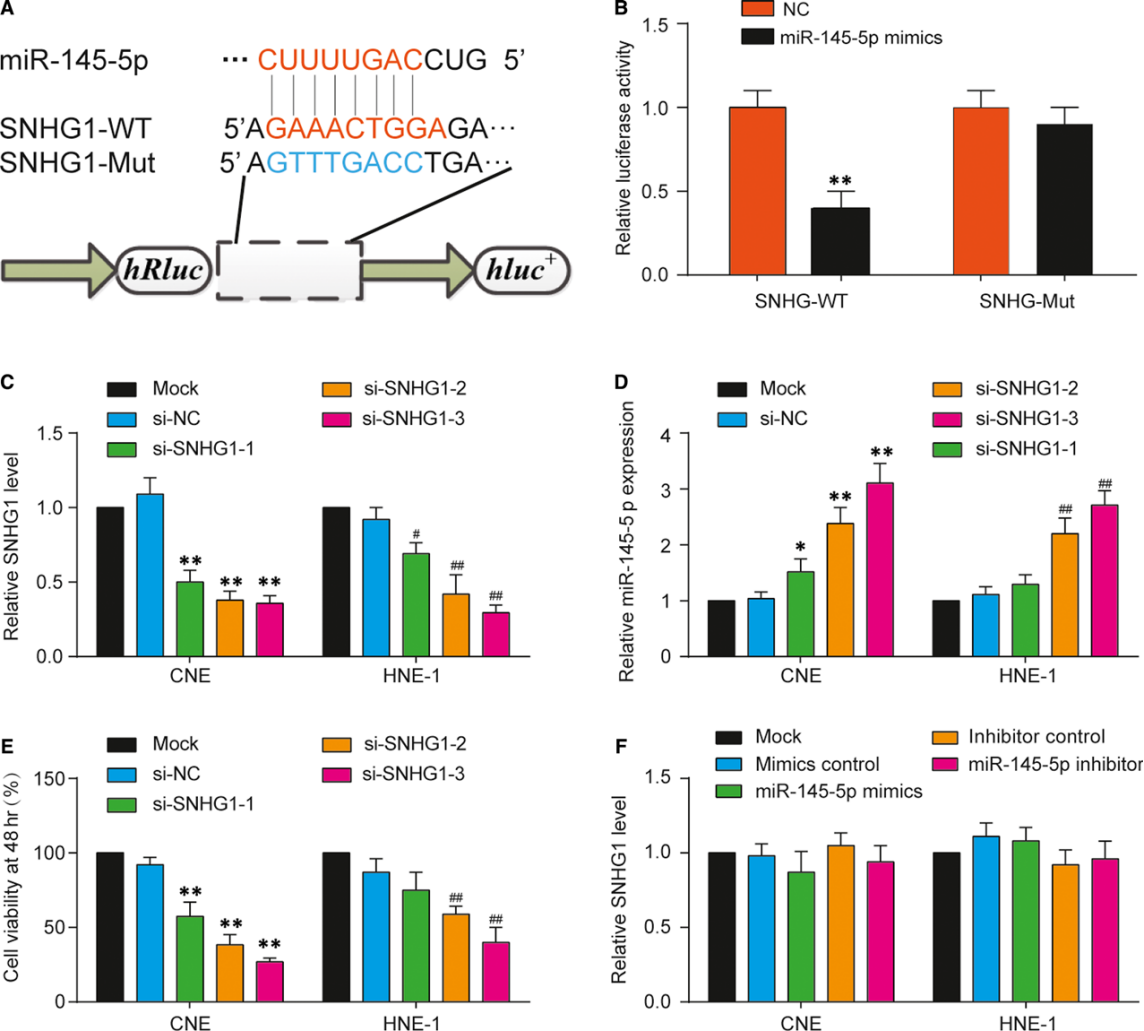
Inhibition of miR‐145‐5p by SNHG1 in nasopharyngeal carcinoma. (**A**) Schematic of luciferase reporter vector constructs SNHG1 wild‐type (WT) and the miR‐145‐5p binding site mutated (Mut) one. (**B**) MiR‐145‐5p evidently suppressed the luciferase activity of the SNHG1 WT but had no obvious effects on the luciferase activity of SNHG1‐Mut. Mean ± S.D. **P < 0.01, compared with NC group. (**C**) The low levels of SNHG1 in CNE and HNE‐1 cells transfected with SNHG1 siRNAs detected by qRT‐PCR. Mean ± S.D. (**D**) The relative expression levels of miR‐145‐5p in CNE and HNE‐1 cells transfected with SNHG1 siRNAs were detected by qRT‐PCR. Mean ± S.D. **P < 0.01, compared with mock group. (**E**) Harvested cells were cultured for 48 hrs, and cell viability was accessed *via* MTT assay. (**F**) The modulation of miR‐145‐5p expression did not affect SNGH1 expression in both cell lines. *P < 0.05, **P < 0.01, compared with mock group.

Using miRDB database blast prediction, we found that SNHG1 had a putative miR‐145‐5p targeting site, which was verified by luciferase reporter assay (Fig. 2A). Subsequently, the simultaneous addition of miR‐145‐5p mimics and SNHG1 WT showed significantly impeded luciferase activity (*P* < 0.01, Fig. 2B). Three SNHG1 siRNAs were designed and transfected into CNE and HNE‐1 cells at the concentration of 50 nM; qRT‐PCR was utilized to measure the expression of SNHG1 at 48 hrs post‐transfection. Si3‐SNHG1 showed the best knockdown efficiency, thus was chosen for further analysis (*P* < 0.01, Fig. 2C). After the knockdown of SNHG1 levels in NPC cell lines CNE and HNE1 using si‐SNHG1, the expression of miR‐145‐5p was significantly up‐regulated compared with the control group (*P* < 0.01, Fig. 2D). Besides of that, MTT assay results showed that Si‐SNHG1‐3 significantly suppressed cell viability (*P* < 0.05, Fig. 2E). miR‐145‐5p overexpression did not affect SNHG1 expression (Fig. 2F). These findings suggested that miR‐145‐5p was the target of SNHG1 and could be suppressed by SNHG1 in NPC.

### MiR‐145‐5p targeted *NUAK1* 3′ UTR and suppressed *NUAK1* expression

**Figure 3:**
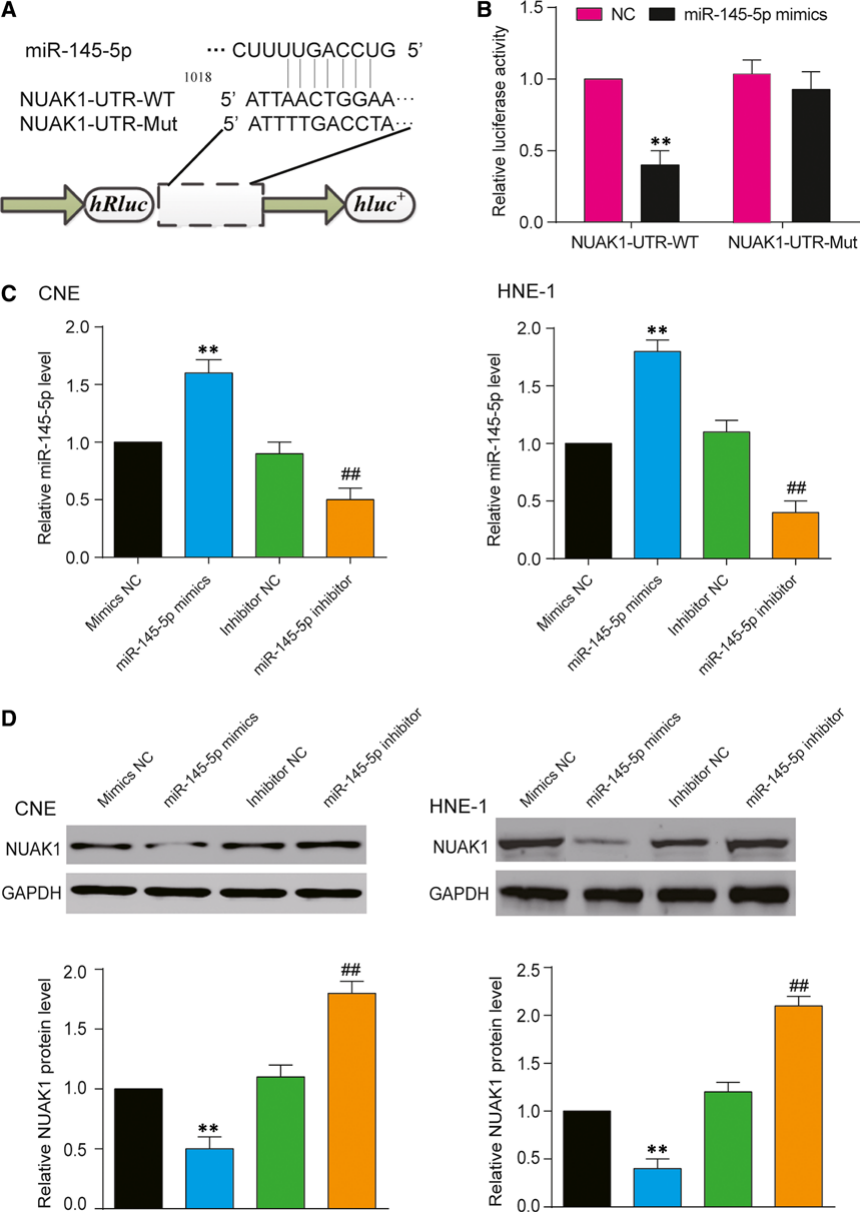
MiR‐145‐5p targeted *NUAK1* 3′ UTR and suppressed *NUAK1* expression. (**A**) Schematic of luciferase reporter vector constructs *NUAK1* 3 ‘UTR wild‐type (WT) and the miR‐145‐5p binding site mutated (Mut) one. (**B**) MiR‐145‐5p evidently suppressed the luciferase activity of the *NUAK1*‐UTR‐WT but had no obvious effects on the luciferase activity of *NUAK1*‐UTR‐Mut. Mean ± S.D. **P < 0.01, compared with NC group. (**C**) QRT‐PCR showed that the relative expression level of miR‐145‐5p in CNE and HNE‐1 cells transfected with miR‐145‐5p mimics increased and that with miR‐145‐5p inhibitors decreased. Mean ± S.D. **P < 0.01, compared with miR‐NC mimics group; ^*##*^ *P* < 0.01, compared with NC inhibitor group. (**D**) Western blot showed that the relative protein level of *NUAK1* in CNE and HNE‐1 cells transfected with miR‐145‐5p mimics decreased and that with miR‐145‐5p inhibitors increased. Mean ± S.D. **P < 0.01, compared with miR‐NC mimics group and NC inhibitor group; ^##^ *P* < 0.01, compared with inhibitor NC group.

Using miRDB prediction, we also identified the predicted miR‐145‐5p targeting site on *NUAK1* 3′UTR (Fig. 3A). Dual‐luciferase reporter assay showed that miR‐145‐5p can notably inhibit the luciferase activity of *NUAK1*‐UTR‐WT (*P* < 0.05, Fig. 3B), indicating *NUAK1* was the target of miR‐145‐5p. The expression of miR‐145‐5p in CNE and HNE‐1 cell lines was significantly increased after overexpression of miR‐145‐5p and notably decreased after the transfection of miR‐145‐5p inhibitor (*P <* 0.01, Fig. 3C). The expression of *NUAK1* in CNE and HNE‐1 cell lines was significantly decreased after overexpression of miR‐145‐5p and notably increased after the transfection of miR‐145‐5p inhibitor (*P* < 0.01, Fig. 3D). The results showed that miR‐145‐5p inhibited the expression of *NUAK1* by targeting *NUAK1* 3′UTR in NPC cells.

### The effects of miR‐145‐5p on NPC cell aggressiveness

**Figure 4:**
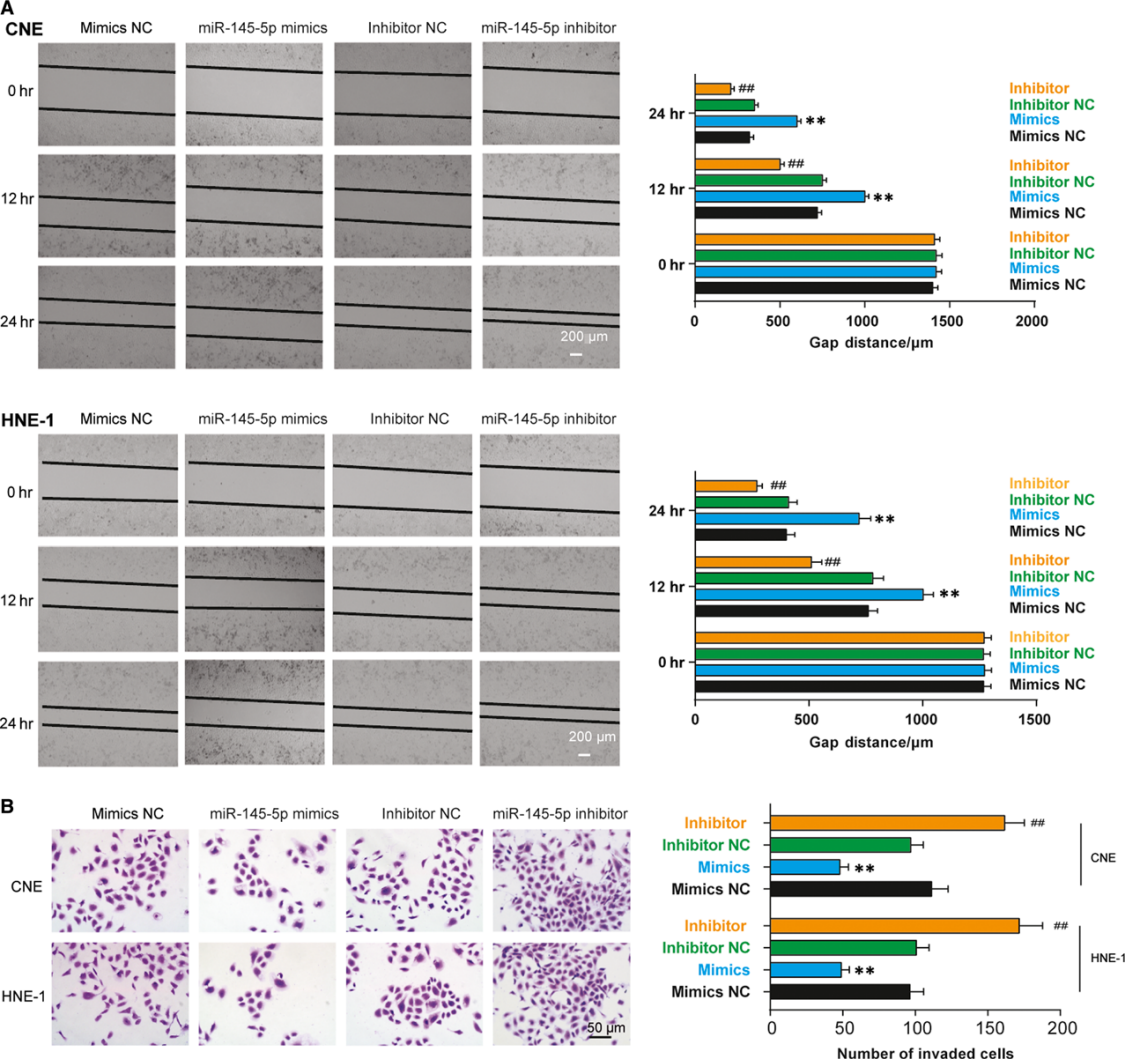
The effects of miR‐145‐5p on NPC cell aggressiveness. (**A**) Within 24 hrs after transfection, CNE and HNE‐1 cells transfected with miR‐145‐5p mimics showed significantly bigger gap distance than those in mimics NC group. On the contrary, the two cell lines transfected with miR‐145‐5p inhibitor showed significantly shorter gap distance than those in inhibitor NC group. Scale bar: 200 μm. (**B**) miR‐145‐5p mimics group had significantly larger number of invaded cells and smaller number of invaded cells compared with the corresponding NC groups. Scale bar: 50 μm. **P < 0.01, compared with miR‐NC mimics group and NC inhibitor group; ^##^ *P* < 0.01, compared with inhibitor NC group.

Within 24 hrs after transfection, CNE and HNE‐1 cells transfected with miR‐145‐5p mimics showed significantly bigger gap distance than those in mimics NC group. On the contrary, the two cell lines transfected with miR‐145‐5p inhibitor showed significantly shorter gap distance than those in inhibitor NC group (Fig. 4A). miR‐145‐5p mimics group had significantly larger number of invaded cells and smaller number of invaded cells compared with the corresponding NC groups (Fig. 4B).

### Down‐regulation of SNHG1 and *NUAK1* expression inhibited the aggressiveness of NPC cells

**Figure 5:**
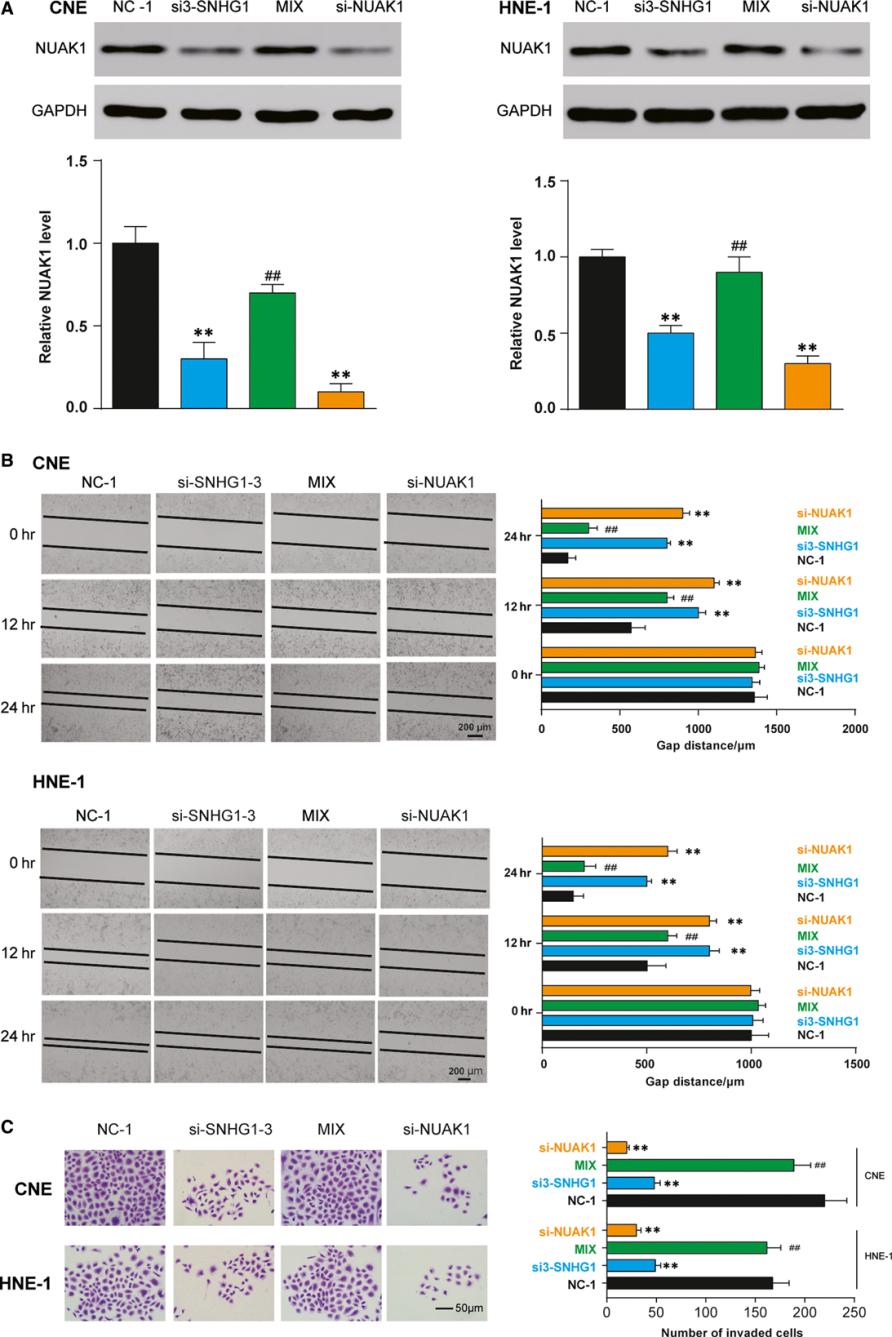
Down‐regulation of SNHG1 and *NUAK1* expression could inhibit the migration and invasion of NPC cells. (**A**) Western blot showed that the relative protein levels of *NUAK1* in CNE and HNE‐1 cells transfected with si3‐SNHG1 and si‐*NUAK1* decreased compared with si‐NC group, and inhibition of miR‐145‐5p overcame the decreased expression of *NUAK1* caused by si3‐SNHG1. (**B**) Wound‐healing assays showed that depletion of SNHG1 and *NUAK1* inhibited cell migration abilities of CNE and HNE‐1 cell lines. However, cotransfection of si3‐SNHG1 and miR‐145‐5p inhibitors could abrogate this inhibition. (**C**) Transwell invasion assay indicated that transfection of si3‐SNHG1 and si‐*NUAK1* inhibited cell invasion ability. However, the inhibitory effects of si3‐SNHG1 could be abolished by cotransfection with miR‐145‐5p inhibitors. Scale bar: 50 μm. Mean ± S.D. **P < 0.01, compared with NC‐1 group; ^*##*^ *P* < 0.01, compared with si3‐SNHG1 group.

In both cell lines, the knockdown of SNHG1 and *NUAK1* led to impeded levels of *NUAK1* in both CNE and HNE‐1 cell lines. The rescue experiments showed that the inhibition of miR‐145‐5p compromised the decreased expression of *NUAK1* caused by si‐SNHG1 (*P* < 0.01, Fig. 5A). Additionally, wound‐healing assays showed that depletion of SNHG1 and *NUAK1* inhibited cell migration of CNE and HNE‐1 cell lines. However, cotransfection of si‐SNHG1 and miR‐145‐5p inhibitors could abrogate this inhibition (*P* < 0.05, Fig. 5B). Furthermore, transwell assay indicated that the transfection of si‐SNHG1 and si‐*NUAK1* inhibited cell invasion. Yet, these effects could be abolished by cotransfection with miR‐145‐5p inhibitors (*P* < 0.05, Fig. 5C). Taken together, these findings implied that SNHG1 could modulate *NUAK1* expression to affect cell migration and cell invasion *via* miR‐145‐5p in NPC.

### The effects of the modulation of miR‐145‐5p, SNHG1 and *NUAK1* on nude mice tumour metastasis and lymph node metastasis

**Figure 6:**
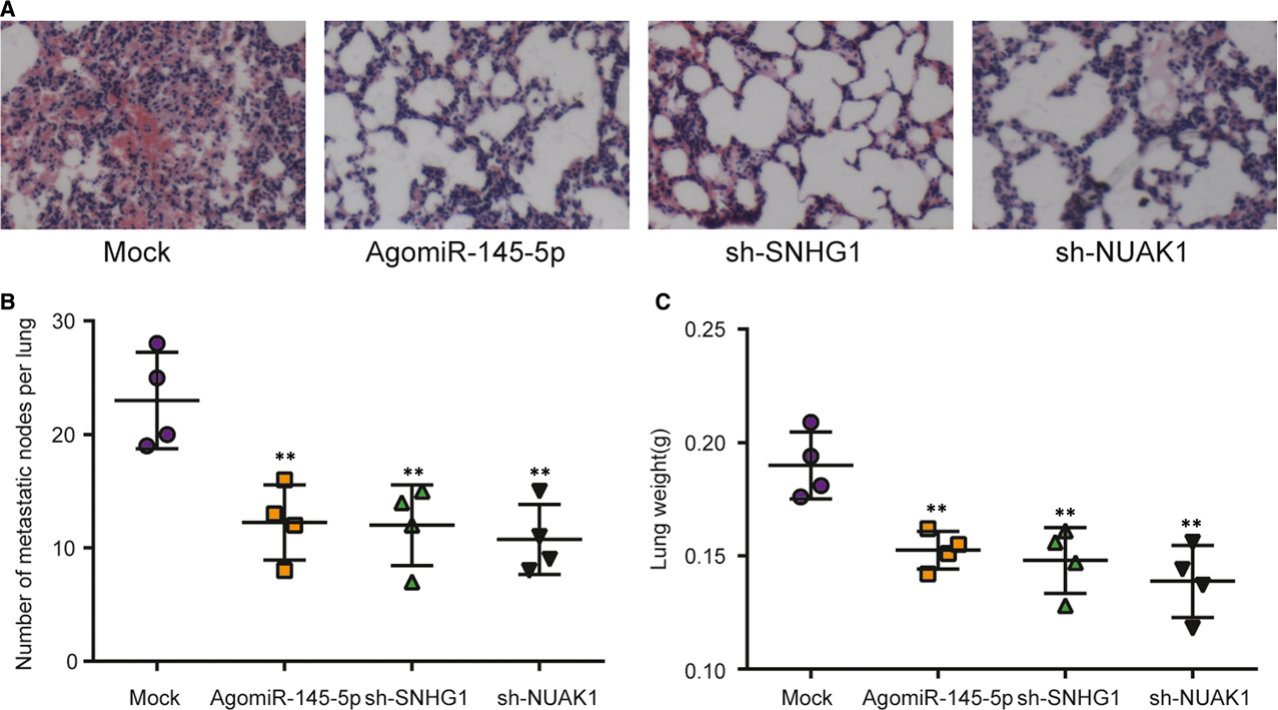
MiR‐145‐5p overexpression and SNHG1 and *NUAK1* knockdown inhibited metastasis in nude mice. (**A** and **B**) Compared with mock group, the number of pulmonary nodules in nude mice agomiR‐145‐5p‐, sh‐SNHG1‐ and sh‐NUAK1 stably transfected cells was reduced. Magnification: 100×. (**C**) After the mice were killed, lung tissues were isolated and weighed. Lung weight was smaller in mice injected with stably transfected CNE cells than injected with mock cells. **P < 0.01, compared with mock group.

The lung metastasis models were established *via* injecting CNE cells at the lateral tail veins. To investigate the influences of SNHG1, miR‐145‐5p and *NAUK1* on the aggressiveness of CNE cells *in vivo*, mice were divided into four groups: injected with untreated cells or cells stably knocked out of SNHG1 or transfected using agomiR‐145‐5p or stably silenced with *NAUK1* shRNA. Compared with mock group, the number of pulmonary tumour nodules in other three groups was all reduced (*P* < 0.01, Fig. 6A and B). The weight of the lungs in the nude mice of experiment group was significantly smaller than that in the mock group (*P* < 0.01, Fig. 6C). Taken together, we confirmed that down‐regulation of SNHG1 or NAUK1 or up‐regulation of miR‐145‐5p inhibited the metastasis ability of CNE cells *in vivo*.

### *NUAK1* inhibition suppressed AKT signalling and EMT (epithelial interstitial transformation)

**Figure 7:**
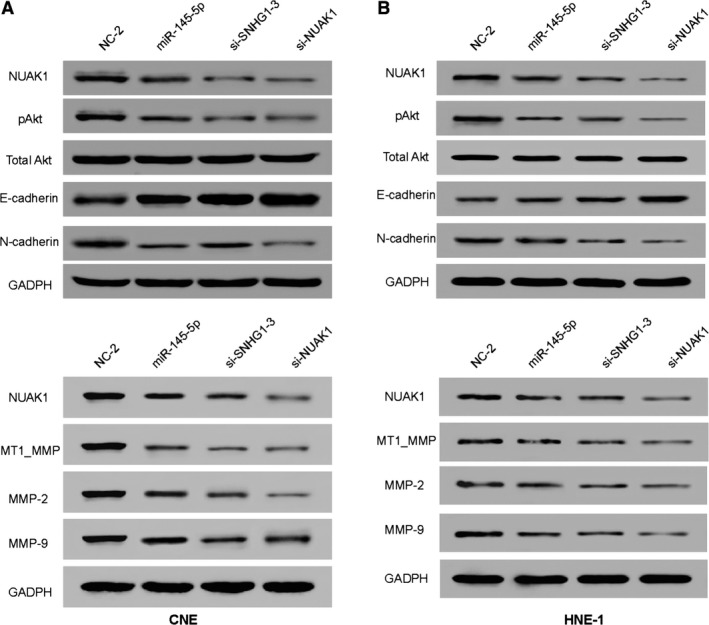
*NUAK1* inhibition suppressed AKT signalling pathway and EMT (epithelial interstitial transformation). (**A** and **B**) Cells were harvested at 48 hrs after transfection. Total protein was collected and analysed *via* Western blot assay. Inhibition of *NUAK1* expression by miR‐145‐5p, si‐SNHG1 and si‐*NUAK1* led to the down‐regulated expression of phosphorylated AKT (p‐Akt), mesenchymal marker N‐cadherin, MMP‐2, MMP‐9 and its regulatory factor MT1‐MMP and the up‐regulated expression of E‐cadherin in CNE cells as well as in HNE‐1 cells.

In this study, phosphorylated but not total Akt, the mesenchymal marker N‐cadherin, MMP‐2, MMP‐9 and its regulatory factor MT1‐MMP were reduced in CNE cells (Fig. 7A) as well as in HNE‐1 cells (Fig. 7B) after transfection of miR‐145‐5p mimics, si‐SNHG1 and si‐NUAK1. The expression of E‐cadherin increased in both cells.

## Discussion

We have demonstrated the high expression of SNHG1 and the low expression of miR‐145‐5p in NPC cell lines. The expression of SNHG1 was negatively correlated with that of miR‐145‐5p. Furthermore, our study demonstrated that SNHG1 suppressed miR‐145‐5p expression and activity *via* direct interaction. We also found that miR‐145‐5p targeted *NUAK1* 3′UTR to inhibit *NUAK1* and that up‐regulation of SNHG1 could promote the expression of *NUAK1*. Therefore, SNHG1 positively regulated *NUAK1* expression by targeting miR‐145‐5p. The knockdown of SNHG1 modulated *NUAK1* expression and impeded the aggressiveness of NPC cells *via* miR‐145‐5p.

Accumulative evidence has demonstrated that SNHG1 plays an essential role in tumorigenesis. Cui *et al*. demonstrated that SNHG1 was up‐regulated in NSCLC tissues and cell lines. *In vitro* experiments showed that high SNHG1 expression was related to larger tumour sizes and advanced TNM stages, and down‐regulation of SNHG1 suppressed cell proliferation in lung cancer 10. Hu *et al*. also found that lncRNA SNHG1 promoted cell proliferation in gastric cancer by regulating *DNMT1* 20. Besides, Sahu *et al*. revealed that the up‐regulation of SNHG1 could act as an independent prognostic biomarker for event‐free survival in neuroblastoma 21. Also, Wang *et al*. indicated that ectopic expression of SNHG1 promoted cell proliferation and cell invasion while reduced cell apoptosis 8. Zhang *et al*. found that SNHG1 might play a promoter role in lung squamous cell carcinoma 22. These are all about the tumorigenesis function of SNHG1 in different cancers. However, the function of SNHG1 in NPC has not been studied till now. Here, for the first time, we verified that SNHG1 exhibited tumour‐promoter effects in NPC by regulating cell invasion and migration. Our study was in accordance with previous evidence and was of crucial significance.

Recent advances in ceRNA field have shown that lncRNAs could interact with miRNAs to exert influence on NPC. Jin *et al*. revealed that MALAT1 regulated cancer stem cell activity and radioresistance through regulating miR‐1/slug axis in NPC 23. Li *et al*. also found that lncRNA H19 interacted with miR‐630 to regulate *EZH2* expression and further promoted cell invasion in NPC 13. Moreover, Lu *et al*. reported that lncRNA NEAT1 could modulate the miR‐204/*ZEB1* axis to regulate EMT phenotype and radioresistance in NPC 24. More importantly, Song *et al*. found that the lncRNA XIST exhibited as a promoter of NPC partially through negatively regulating miR‐34a‐5p and then activating E2F3 functioning 25. These studies indicated that lncRNAs could regulate miRNAs to exert influence on cancer development. It was shown in the present study that miR‐145‐5p functioned as a tumour suppressor, whereas SNHG1 reversed the antitumour effect of miR‐145‐5p by direct modulating miR‐145‐5p, thus promoting cell propagation and aggressiveness. Our results were consistent with the previous studies.

*NUAK1* has been found in many studies to act as the target of miRNAs in during the development of neoplasms. For example, Obayashi *et al*. reported that miR‐203 impeded the aggressiveness and EMT induction of head and neck squamous cell carcinoma by targeting *NUAK1* 26. Similarly, it was announced by Xiong *et al*. that miR‐145 inhibited intrahepatic cholangiocarcinoma (ICC) proliferation by targeting *NUAK1* and its downstream effectors, demonstrating significance in clinical diagnosis and targeted therapy of ICC 16. Besides, Shi *et al*. found that miR‐204 suppressed NSCLC invasion by targeting and down‐regulating *NUAK1* expression 18. Consistently, in this study, we demonstrated that miR‐145‐5p inhibited *NUAK1* expression *via* regulating *NUAK1* 3′ UTR activity, inhibiting the invasion and migration of NPC cells.

Limitation existed in the study, for example, we have only investigated the cancer‐related AKT signalling pathway. Further studies can be carried out on other signalling pathways and their relationship with SNHG1/miR‐145‐5p/*NUAK1* regulation.

To sum up, we concluded that SNHG1 could negatively regulate miR‐145‐5p to up‐regulate *NUAK1* expression, thus promoting NPC cell migration and invasion.

## Conclusion

In conclusion, we found SNHG1 negatively regulated miR‐145‐5p expression, increased *NUAK1* expression and promoted NPC cell invasion and migration. Our findings suggested that the SNHG1/miR‐145‐5p/*NUAK1* axis played a crucial role in NPC and could serve as a critical target for therapy.

## Conflict of interest

The authors declare that there is no conflict of interest.
